# Understanding the preference for homebirth; an exploration of key barriers to facility delivery in rural Tanzania

**DOI:** 10.1186/s12978-017-0397-z

**Published:** 2017-10-17

**Authors:** Fabiola Moshi, Tumaini Nyamhanga

**Affiliations:** 1 Department of Clinical Nursing, University of Dodoma, Dodoma, Tanzania; 2 Department of Developmental Studies, Muhimbili University of Health and Allied Sciences, P.O BOX 65004, Dar es Salaam, Tanzania

**Keywords:** Home childbirth, Health facility, Pregnancy complication, Place of birth, Sumbawanga, Rural Tanzania

## Abstract

**Background:**

There is limited information on the effect of expectant parents’ socio-cultural perceptions and practices on the use of skilled birth attendants (SBAs) in rural Tanzania. The purpose of this study was to explore the socio-cultural barriers to health facility birth and SBA among parents choosing home birth in rural Tanzania, specifically in the Rukwa Region.

**Methods:**

This study used a descriptive exploratory methodology. Purposive sampling was used to recruit study participants for both in-depth interviews (IDIs) and focused group discussions (FGDs). Qualitative research methods, including FGDs and IDIs, were utilized in data collection. The respondents were men and women whose youngest child had been born at home within the prior 12 months. A thematic approach was used for data analysis.

**Results:**

The main themes that emerged regarding barriers to the use of health facility were 1) limited decision-making by men on place of delivery; 2) low risk perception by men and its interference with health facility birth; 3) men’s limited resource mobilization for health facility birth and 4) females’ perceptions that pregnancy and childbirth are low-risk events.

**Conclusion:**

This qualitative study demonstrates that apart from well-documented structural barriers to skilled birth attendance in rural Tanzania, the low risk perception among both men and women plays a substantial role. The low risk perception among both men and women affects the use of SBAs in two ways. First, women become negligent and take risk of delivering at home. Second, male partners do not seriously mobilize resources for health facility childbirth. These findings reinforce the urgent need to implement creative programs to increase genuine male participation in facilitation of health facility childbirth.

## Plain English summary

Health facility birth is a key strategy for improving birth outcomes in developing countries. Both parents’ perceptions of the risks associated with pregnancy and childbirth may influence their choice regarding place of childbirth, although these perceptions may operate in different contexts. Male partners’ risk perceptions and their social roles/responsibilities in pregnancy and childbirth influence each other. What male partners are likely to do or not do (social roles) in combination with safe motherhood is partly determined by gender attitudes and beliefs. Likewise, female partners’ obstetric risk perceptions may be influenced by the socio-economic and cultural circumstances in which they live, including the supportive or non-supportive roles of their husbands/male partners. All of these interrelated factors ultimately determine the couple’s level of health facility birth preparedness, which then defines the choice of place of delivery.

Thus, in summary, there are two pathways. The first one is low obstetric risk perception, which leads to low levels of health facility birth preparedness and is likely to result into home delivery. Here, an unskilled person (traditional birth attendant [TBA] and/or relative) attends to the mother. The second is high obstetric risk perception, which leads to high levels of health facility birth preparedness and is likely to result in health facility delivery (skilled attendants).

## Background

Maternal mortality remains an urgent public health challenge worldwide. The high maternal mortality ratio of 216/100,000 live births worldwide per year is not acceptable [1]. The overwhelming majority of all maternal illnesses and deaths worldwide occur in sub-Saharan Africa and southern Asia. In 2013, these two regions alone accounted for 86% of the global burden for maternal deaths [2]. The maternal death proportion in developing countries is approximately 14 times higher than in more developed regions [2]. A woman in the global South has a lifetime risk of maternal death of 1 in 76, compared to 1 in 10,000 in the global North. The risk is even greater for Tanzanian women, whose lifetime risk of maternal death is one in forty-four [3]. Tanzania is among the countries with the highest maternal mortality rates in the world, with an estimated Maternal Mortality Ratio (MMR) of 432/100,000 [4].

In addressing this issue as a matter of urgency, the Tanzanian government began implementation of *One Plan*, the *National Road Map Strategic Plan to Accelerate Reduction of Maternal, Newborn and Child Deaths in Tanzania* in 2008. The goal of *One Plan* was to reduce MMR by two-thirds by 2015, in accordance with national goals for meeting the Millennium Development Goals (MDGs). A key target for *One Plan* was to increase coverage of skilled birth attendance (SBA) from 46% to 80%, due to the Ministry of Health and Social Welfare’s identification of low utilization of SBA and health facilities for childbirth as a major cause of maternal and neonatal morbidity and mortality in Tanzania [5]. Following the implementation of *One Plan*, 90% of all pregnant women managed to attend antenatal care at least once during their pregnancy, but only 43% of women managed to attend four times or more.

However, only 51% of women in Tanzania received skilled delivery care at available health facilities in 2010 [6]. In that same year in the Rukwa Region of Tanzania, it was found that 97% of women attended the antenatal clinic at least once during their pregnancy [6]. However, this region still had the highest percentage of home delivery in Tanzania, with 69.6% of women delivering at home with unskilled attendants [6]. This finding arose despite the fact that many districts in Rukwa Region were equipped with several health facilities – an average of one dispensary for two villages.

While the average maternal mortality rate in Tanzania in 2012 was 432 deaths per 100,000 live births, Rukwa Region had the highest in the country, with a maternal mortality ratio of 860 deaths per 100,000 live births according to the National Bureau of Statistics [4]. Likewise, the national overall infant mortality rate was estimated to be 46 deaths per 1000 live births, but Rukwa Region had an infant mortality rate of 54.8 deaths per 1000 live births [4]. Given these findings, the risk of complication during childbirth is unpredictable; however, it is questionable why such a high percentage of women fail to access skilled attendance during delivery.

Health facility birth preparedness has the potential to reduce all three phases of delays to access maternal services. These delays include delay in decision-making to seek healthcare, delay in reaching a health facility and delay in obtaining appropriate care upon reaching a health facility [7]. These delays impact access to skilled birth attendants.

To improve maternal services utilization, the involvement of male partners in mobilizing resources for health facility birth must be a focus for intervention [8]. Empowering men with necessary information about emergency obstetric conditions as well as engaging them in birth preparedness is vital to improving maternal services utilization, Iliyasu et al. [9].

Previous studies examining factors that hinder access to skilled birth attendants have focused on access to supplies and social economic factors, including the cost of care, level of acceptance, affordability, and poor quality of care in health facilities [10, 11]. However, there is a dearth of information about individuals’ socio-culturally motivated perceptions and practices. This paper presents findings of a descriptive exploratory study focused on understanding the socio-cultural beliefs and practices that compromise access to skilled birth attendance in Sumbawanga District in Rukwa Region.

## Methods

### Study site

The study was conducted in the rural setting of Sumbawanga District in Rukwa Region, Tanzania from March to April 2012. The district has a population of 305,846 [12]. Sumbawanga District has 120 health facilities, including 10 health centres with the capacity for providing basic emergency obstetric care and 2 health centres with the capacity for providing both basic emergency obstetric care and comprehensive emergency obstetric care. The district also has 108 dispensaries with the capacity for addressing mother and child health concerns and conducting normal deliveries. These health centres, which can provide both basic and comprehensive emergency obstetric care, are the first line of referral to dispensaries and health centres without the capacity for providing comprehensive emergency obstetric care. The quality of care in these primary health care centres is also questionable. A study assessing the quality of care in primary health services in Tanzania reported a poor quality of care, especially for poor rural communities [13]. The district did not have a district hospital, but all referrals were taken care of by Sumbawanga Regional Hospital, which represents the secondary referral level for emergency obstetric care and provides services 24 h a day. It is 90 km from Mtowisa, one of the health centres that provides comprehensive emergency obstetric care to Sumbawanga Regional Hospital. In 2012, when this study was conceptualized, the percentage of deliveries that occurred in health facilities in Rukwa Region (in which Sumbawanga rural district belongs) was only 29.5% [6]. Although there is considerable evidence suggesting that facility births are increasing markedly in many parts of the country [14], it is important to understand socio-cultural barriers in the rural settings, where changes may be realized in a relatively slower manner. Sumbawanga, like other rural districts, is located in a remote area, with deeply rooted traditions that require context-specific and evidenced-based interventions.

### Study design

This was a descriptive exploratory study. Qualitative research methods were employed, specifically in-depth interviews and FGDs. The respondents were matched couples: partnered men and women whose youngest child had been delivered at home less than 12 months prior.

### Sample size and sampling process

A purposive sampling technique was employed using the criteria of high proportions of home deliveries. The divisions in Sumbawanga District were listed, and two out of seven geographic divisions were purposefully selected, namely, Kipeta and Mwimbi. The two selected divisions had the highest number of home deliveries. The twelve wards in Kipeta were listed, a random sampling technique using lottery was performed, and Ilemba ward was selected. The ten wards of Mwimbi were listed, a random sampling technique using lottery was performed, and Mwimbi ward was selected. All villages from each ward were listed, and two villages from each ward were picked randomly. The four randomly selected villages were Ilemba, Selengoma, Mwimbi, and Musoma. From the villages, 16 households were identified in which a woman had given birth at home or at the traditional birth attendant’s home. These households were conveniently selected. The male and female partners were both recruited for participation by principal investigator and village health workers. The village chairpersons were informed and allowed data collection to occur in their village; participants were then recruited conveniently. A total of 32 in-depth interviews and four FGDs were conducted across the four villages. Since the data were being quickly analysed as they were being collected, the research team noticed that no new information emerged after completing 32 interviews and 4 FGDs, as similar views were recurring over and over again. That is, saturation was achieved with that sample size, and data collection stopped at that point.

### Inclusion and exclusion criteria

The inclusion criteria of this study were being parents of children 0–12 months (living or dead) who were born through home birth, living together as a couple from pregnancy until the time of selection, and consenting to participate.

The exclusion criteria to this study were being single parents, being parents of 0–12 months infants born in health facilities, being parents of children aged more than 12 months who were born through home birth, being parents who met the inclusion criteria but refused to participate, and being parents who met the inclusion criteria but one of the partners was not mentally fit to be interviewed.

### Data collection methods

Focused group discussions (FGDs) and in-depth interviews (IDIs) were used to collect data, using two slightly different semi-structured question guides. The guide for FGDs was focused on exploring general socio-cultural barriers prevailing in the community, whereas the guide for IDIs referred to personal experiences with home childbirth. The FGD and interview guides were developed in English and then translated into Kiswahili to facilitate their use in the field. The same participants were used for both FGDs and IDIs. Data collection began with FGDs, followed by IDIs; that is, after a general exploration of questions in group discussions, the researchers went in greater depth by interviewing individuals one-on-one to understand how said socio-cultural barriers impinge on the uptake of real-world health facility delivery.

With respect to FGDs, men and women participated in separate discussion groups. These discussions provided an opportunity for the researchers to explore the socio-cultural context of home deliveries. The interaction among participants in an FGD provided an opportunity for participants to talk about perceptions motivated by the prevailing socio-cultural beliefs and practices around pregnancy and delivery in the community. The IDIs were conducted with both men and women, and the interviewees’ spouses or partners were not present during the interview. These interviews focused on investigating personal perspectives of risk perception related to pregnancy and personal experiences of home delivery.

The FGDs and interviews were conducted by the lead author, while a trained research assistant operated the tape recorder and took notes. After self-introductions, we used an African style of greeting as an ice breaker to create a comfortable atmosphere. This process involved asking simple questions about wellbeing of the family, such as the following: how is the little one doing? How about the spouse? How about the other siblings, if any? Then, the first substantive question was as follows: how is the health of the mother and children? This was followed by more specific questions on childbirth services and related socio-cultural circumstances.

FGDs took 50–60 min each, while in-depth interviews took 40–45 min each. The lead author conducted the interviews, and a trained research assistant operated the tape recorder and took notes.

### Ethical considerations

The ethical clearance for this study was obtained from Muhimbili University of Health and Allied Sciences (MUHAS). The prospective FGD participants and key informants were requested to participate in the study. Before consenting, the moderator told the participants that their participation was purely voluntary. Furthermore, they were informed that no names were required and that data would be treated with a high level of confidentiality. Moreover, participants were also requested to maintain confidentiality by not revealing personal experiences that may feature in discussions with other people who did not participate. Finally, each potential participant was informed of his or her right to refuse to participate. Participants consented by signing an informed consent form.

### Data analysis

Tape recorded data were transcribed verbatim and translated from Kiswahili to English. The data were analysed using a thematic approach in three stages [15, 16]. The first stage consisted of line-by-line coding of field notes and transcripts. That is, each sentence of text was coded (i.e., assigned a word or phrase) according to the meaning it conveyed. In the second stage, the authors examined the codes generated for similarities and differences. Codes that conveyed similar meanings were grouped together and assigned a phrase that portrayed the common message. These phrases for several groups of codes constituted descriptive themes. The third stage involved a higher level of interpretation, whereby analysis went beyond the content of the raw data towards answering the main research question. That is, authors inferred barriers to health facility childbirth from the respondents’ views captured in descriptive themes – a process which resulted in more abstract analytical themes. Data analysis was completed by the two authors separately. They then met, compared outputs, and achieved a consensus on the themes.

In this study, the realistic value of findings was determined using triangulation; both IDIs and FGDs were used. A member checking technique was utilized during data collection to increase the trustworthiness of the findings. This technique involved the researcher restating, summarizing, or paraphrasing the information received from FGD or key informant to ensure that what was heard or written down was correct. In this way, the researcher could be confident that the qualitative findings of this study were logical and grounded in the data.

## Results

### Socio-demographic characteristics

A total of 32 men and women (16 couples) participated in the study. The average age of males in the study was 37 years (age range 24–62) and the average age of females was 31 years (age range 21–39). The majority of respondents were multipara, i.e., had more than three children. The distance from home to the nearest health facility was less than two kilometres for the majority of couples interviewed. Table 1 represents the socio-demographic characteristics of the men and women who were involved in the study.

**Table 1:** Demographic characteristics of participants (*n* = 32)

Characteristics	Men	Women
Age (years)		
20–29 years	4	6
30–39 years	8	10
> 40 years	4	0
Education		
None (never attended school)	0	1
Primary	16	15
Secondary	0	0
College	0	0
Distance to Nearest Health Facility		
< 2 Kilometres	10	10
> 2 Kilometres	6	6
Occupation		
Peasants	13	15
Small business	2	1
Other	1	0
Religion		
Christian	14	14
Muslim	2	2
Other	0	0

### Study themes

**Fig. 1:**
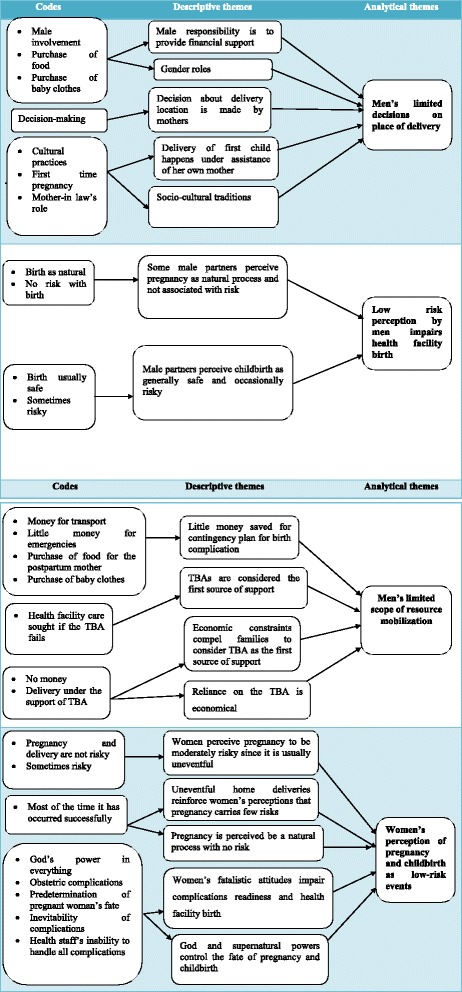
Thematic Network: from codes to analytical themes

Male and female interviews were collected separately and then analysed separately. The analyses were done separately because men and women have different perceptions and experiences regarding childbirth. Although both seemed to have low risk perceptions, the bases were different. Four main themes emerged from the thematic network analysis process (Fig. 1): 1) Men’s limited decisions on place of delivery; 2) Low risk perception by men and its interference with health facility birth; 3) Men’s limited scope of resource mobilization for health facility birth and 4) Women’s perception of pregnancy and childbirth as low-risk events. These four main themes will serve as key sub-headings in the subsequent sections. These four themes were the outcome of thematic analysis, and none of them were predetermined before actual data analysis.

1. Men’s limited decisions on place of delivery
  In-depth interviews and focused group discussions revealed that men in this region of Tanzania had limited involvement in decision-making about the location of delivery. Participants identified two socio-cultural norms associated with men’s limited participation in this realm of decision-making: 1) tradition and 2) birth location decision-making as a woman’s domain.
  In terms of tradition, the participants explained that men hold a belief in the tradition that requires that all married women return to their parents’ home for their first delivery. Within this cultural practice, it is believed that there is simply no room for men to intervene due to the tradition.
*Fathers do not have a say when it comes to the decision about the place of delivery. The decision is usually made by mothers and mothers-in-law. For example, in my case, during her first pregnancy, my wife went to her mother for delivery—for it is a tradition for the first delivery to take place at her parents’ home.*
*(24-year-old male, Ilemba Village, FGD)*
  It was revealed that gender roles surfaced as a major influencing factor in the decision on delivery location. The study participants explained repeatedly that male partners are less concerned about deciding the place of delivery because it is believed to be the role of women to decide the birth location. Meanwhile, men perceived their role to be one of providing crucial financial support.
*The decision maker on the place of delivery is the woman. The husband should provide material support, like a hen for soup after a baby is born, or a bar of soap as a gift for the traditional birth attendant. (29-year-old male, Mwimbi Village, FGD)*
2. Men’s low risk perception impairs health facility birth
  Male respondents overwhelmingly perceived pregnancy and childbirth as a natural process that commonly produces safe outcomes. A participant in an in-depth interview said that men consider childbirth as an ordinary rite of passage:
*Pregnancy and childbirth are not risky because pregnancy and childbirth are a part of becoming an adult and having a baby is normal.*
*(25-year-old male, Mwimbi Village, IDI)*
  In addition, both male and female respondents reported that they did not have a habit of discussing the logistics of the delivery with their spouse. Men cited their busy schedules and childbirth as a natural and uneventful process as the primary reasons for not having such discussions. A 24-year-old from Ilemba Village explained:
*We are not used to discussing how delivery will be carried out. We are busy looking for money and we take it for granted that childbirth is a normal and natural process that will just happen. (24-year-old male, Ilemba Village, FGD)*
3. Men’s limited scope of resource mobilization
  None of the participants identified health facility delivery as a priority; in fact, health facility delivery was regarded as a second option. Home delivery was universally preferred among the study participants.
  Some respondents mobilized resources during pregnancy in preparation for a complication by focusing on transporting their female spouse to the health facility. Indeed, some of the male participants argued eloquently that there was no need to save money, given that childbirth assisted by TBAs is inexpensive, readily available and of good quality. They also explained that the TBAs generously provided services, and the payment for the service could be made following the home birth delivery.
*We normally do not prepare for delivery because traditional birth attendants are available all the time. They are there, and when labour starts we call our mothers-in-law and they are the ones who look for traditional birth attendants. The traditional birth attendants are experienced and they know how to assist delivery.*
*(33-year-old male, Mwimbi Village, FGD)*
  The secured resources were not utilized for ensuring that a delivering woman has access to the skilled birth attendant at a health facility. Instead, the money was reserved to be spent only if a complication occurred while the woman was under the care of TBAs.
*I kept some money to assist with transport if a delivery under the support of the TBA developed any complication. I saved about 50,000 Tanzanian Shillings [25 USD]. I was sure the money would be enough for transport.*
*(34-year-old male, Selengoma Village, IDI)*
  Many study respondents viewed resource mobilization not as a strategy for covering delivery-related costs, but rather as a strategy to cover the costs of food and clothing after the baby’s arrival. Men described an understanding of both the need to save money and resource mobilization as one of their key roles in pregnancy participation. Rather than preparing for birth and complication readiness, men were universally focused on assembling financial resources for the most basic needs of the family unit:
*I kept some money to assist me in the delivery for buying meat, Orange [a drink] and soda. Meat is for preparing soup as giving thanks to the traditional birth attendant for the service. (36-year-old male, Musoma Village, IDI)*
*Saving some money is my key responsibility. For this pregnancy, I saved as much as 20,000 Tanzanian Shillings [10 USD] for food and clothing to be used when our baby is born. (32-year-old male, Musoma Village, IDI)*
4. Women’s perception of pregnancy and childbirth as low risk
  The perceived risks of women associated with pregnancy and childbirth were similar to those of men. Following analysis, it emerged that women participated in this study had low levels of risk perception. Their understanding of pregnancy and childbirth as low-risk events were supported by personal experiences of uneventful home deliveries. Meanwhile, women did express a fatalistic belief that complications or bad outcomes associated with pregnancy and childbirth are beyond human control. Within this cohort of women, the experiences of uneventful home delivery appeared to reinforce their perception that pregnancy and childbirth carry low or no risk. In contrast, they viewed pregnancy and childbirth as normal and natural processes.
  Most of the participants had more than one child, and some women had as many as seven children, all delivered safely at home. Their experiences with safe home deliveries assisted by traditional birth attendants influenced their perception of pregnancy and childbirth as safe and without risk. This perspective was echoed by a 30-year-old female participant from Musoma Village:
*Pregnancy and delivery are not risky because most of the time they have occurred successfully. As you can see, this is my seventh child and I have not encountered any problems, not during my pregnancies or during delivery. (30-year-old female, Musoma Village, IDI)*
  The focus group discussions and individual in-depth interviews provided the researchers with an opportunity to explore women’s perceptions of risks and complications as related to pregnancy and childbirth. Several women shared their belief that obstetric outcomes are determined by supernatural powers. They described birth outcomes as something that humans cannot control, believing that good or bad outcomes may occur regardless of precautions or steps taken. This perspective surfaced as a clear reason why women chose to deliver at home and was also identified as a barrier that prevents women from engaging in birth preparedness and complication readiness.
*It is difficult to prevent complications which may happen during pregnancy and childbirth. If it has to happen, it must happen. That is why there are women who go to the hospital and develop complications and sometimes lose their babies. Just as there are women who deliver at home and develop complications and lose their babies.*
*(27-year-old female, Ilemba Village, FGD)*

## Discussion

This study found that many male partners perceive pregnancy and childbirth as normal processes that are not associated with risk. This local understanding does not match the numerous documented risks that are associated with the pregnancy and delivery as well as the postpartum period in Tanzania [17, 18]. For male participants in this study, their positive personal experiences outweighed their understanding of the possible risks. Such perceptions have affected the involvement of male partners in supporting their spouses in accessing delivery services from skilled attendants. Similar findings have been reported in Kenya, Nigeria and Tanzania [9, 18, 19].

This study also found that women who choose to deliver at home are motivated by a history of uneventful previous deliveries. Those who have successfully delivered their babies at home have a low risk perception of home deliveries with TBAs. This finding implies that successful home deliveries lead women to develop trust in TBAs such that some women choose home deliveries because they feel they are not at risk. Similar findings have been reported by Bedford et al. [20]. Health education programmes for maternal and newborn health should emphasize the uniqueness of each pregnancy and that sometimes, obstetric complications may occur unpredictably.

Apart from a personal history of uneventful home deliveries, another factor influencing home birth was a fatalistic attitude that obstetric complications and bad outcomes are pre-determined by supernatural powers. Similar findings have been reported elsewhere in sub-Saharan Africa, in which a review conducted by Bohren et al. [21] showed that some women had a sense of fatalism, as they believed that delivery complications were beyond their control. This perspective may be a direct reflection of lack of confidence in the competence of rural public health system arising from the negative experiences [18, 22]. This study found that socio-cultural traditions limit male partners’ involvement in childbirth location decision-making. Study participants shared that there is a local tradition which requires that married women should return to their parents’ home to give birth to their first child. This local tradition provides social and cultural permission for men’s exclusion from key areas of decision-making around birth preparedness and complication readiness. This finding about exclusion of men from decision-making about the delivery location has been echoed by many other studies that have also found that cultural traditions and beliefs within the household influenced decision-making around childbirth practices and place of delivery [18, 23].

Gender-based division of roles was another factor that affected limited male involvement in decision-making regarding the place of delivery. Male participants in this study saw themselves as responsible for basic financial support for the essentials of transport, clothing and food and are less concerned with child birth arrangements - including childbirth locations which are handled by women themselves. While it may seem reassuring that women have control over childbirth location decision-making, the reality for most rural Tanzanian women is that men retain control over family income. As such, practically, men serve as the gatekeepers, in control of the family income and therefore the delivery location [9]. It follows that limited involvement of men in deciding the place of delivery might reflect a strategy of avoiding financial responsibility for accessing skilled services with SBAs at health facilities. This interpretation is supported by findings in Mali reported by White et al. [24].

Through careful planning and community health messaging, men and women both need to appreciate the reality that home birth remains a risky undertaking in rural Tanzania [25].

This study has some limitations, as it utilized only qualitative methods and purposive selection of the study sample. This approach allows for limited generalization of study findings. However, like most qualitative studies, the goal of this study was not to generalize but rather to provide a deeper contextualized understanding of birth preparedness and complication readiness through the intensive study of home birth parents.

## Conclusion

This qualitative study demonstrates that apart from well-documented structural barriers to skilled birth attendance in rural Tanzania [11, 22, 26, 27], the low risk perception of pregnancy and childbirth among both men and women plays a substantial role. This low risk perception among both men and women affects the use of SBAs in two ways. First, women become negligent and take the risk of delivering at home. Second, male partners do not seriously mobilize resources for health facility childbirth. These findings reinforce the urgent need to implement creative programmes to increase genuine male participation in the facilitation of health facility childbirth. In essence, in order to increase the number of health facility childbirths, there is need to develop interventions that will take the following strategies into account: an education programme to enable men and women unlearn gender norms that promote low risk perception on pregnancy and childbirth; a community based programmes for encouraging joint decision-making between spouses on safe childbirth; and empowering individuals and communities with knowledge on the importance of skilled birth attendance. Community health workers are key to implementation of these strategies.

## Abbreviations

ANC: Antenatal care
FGD: Focused group discussion
HBM: Health belief model
IDI: In-depth interviews
MDG: Millennium development goal
MMR: Maternal mortality ratio
SBA: Skilled birth attendant
TBA: Traditional birth attendant

## References

[1] World Health Organization. Trends in maternal mortality: 1990 to 2015 estimates by WHO, UNICEF, UNFPA, World Bank Group and the United Nations population division [internet]. Geneva: WHO Library Cataloguing-in-Publication Data; 2015. Available from: http://apps.who.int/iris/bitstream/10665/193994/1/WHO_RHR_15.23_eng.pdf. Accessed Aug 2016.

[2] United Nations. The Millennium Development Goals Report [Internet]. United Nations. 2015. Available from: http://www.un.org/milleniumgoals/pdf/pdf/MDG%20rev%20(July%201).pdf. Accessed Aug 2016.

[3] WHO. Trends in Mternal Mortality: 1990–2013. Estimates by WHO,UNICEF, UNFPA, The World Bank and the United Nations Population Division [Internet]. World Health Organisation. 2014. Available from: http://apps.who.int/iris/bitstream/10665/112682/2/9789241507226_eng.pdf?ua=1. Accessed Aug 2016.

[4] National Bureau of Statistics. Mortality and Health [Internet]. Dar es Salaam; 2015. Available from: http://www.mamaye.or.tz/sites/default/files/Mortality_and_Health_Monograph-Tanzania. Accessed Aug 2016.

[5] MoHSW. Tanzania Health Sector Strategic Plan 2015–2020 (HSSP IV). Vol. 2020. 2015. http://www.tzdpg.or.tz/fileadmin/documents/dpg_internal/dpg_working_groups_clusters/cluster_2/health/Key_Sector_Documents/Induction_Pack/Final_HSSP_IV_Vs1.0_260815.

[6] National Bureau of Statistics. Tanzania Demographic and Health Survey [Internet]. Dar es Salaam, Tanzania; 2010. Available from: https://dhsprogram.com/pubs/pdf/FR243/FR243%5B24June2011%5D.pdf. Accessed Aug 2016.

[7] Teela KC, Mullany LC, Lee CI, Poh E, Paw P, Masenior N, et al. Community-based delivery of maternal care in conflict-affected areas of eastern Burma: perspectives from lay maternal health workers. Soc Sci Med. 2009;68(7):1332–40. Available from: http://dx.doi.org/10.1016/j.socscimed.2009.01.033. 10.1016/j.socscimed.2009.01.033 19232808

[8] Martin LT, McNamara MJ, Milot AS, Halle T, Hair EC (2007). The effects of father involvement during pregnancy on receipt of prenatal care and maternal smoking. Matern Child Health J.

[9] Iliyasu Z, Abubakar IS, Galadanci HS, Aliyu MH (2010). Birth preparedness, complication readiness and fathers’ participation in maternity care in a northern Nigerian community. Afr J Reprod Health.

[10] Nwokocha EE (2007). Maternal crises and the role of African men: the case of a Nigerian community. Etude la Popul Africaine.

[11] Mpembeni RN, Killewo JZ, Leshabari MT, Massawe SN, Jahn A, Mushi D (2007). Use pattern of maternal health services and determinants of skilled care during delivery in southern Tanzania: implications for achievement of MDG-5 targets. BMC Pregnancy Childbirth.

[12] National Bureau of Statistics. Basic Demographic and Socio -Economic Profile. Basic Demogr Socio- Econ Profile. 2014. https://tanzania.go.tz/egov_uploads/documents/TANZANIA_MAINLAND_SOCIO_ECONOMIC_PROFILE_sw.pdf.

[13] Leonard KL, Masatu MC. Variations in the quality of care accessible to rural communities in Tanzania. Health Aff. 2007;26(3):w380-92. Available from https://www.ncbi.nlm.nih.gov/pubmed/17389635. 10.1377/hlthaff.26.3.w380 17389635

[14] Van Rijsbergen B . Skilled Birth Attendance in the Tanzanian Lake Region. 2011. https://docgo.org/download/documents/skilled-birth-attendance-in-the-tanzanian-lake-region-a-studyon-women-s-preferences-for-obstetric-care-facilities.

[15] Attridge-Stirling J. Thematic networks: an analytic tool for qualitative research. Qual Res. 2001;1(3):385–405. Available from: http://utsc.utoronto.ca/~kmacd/IDSC10/Readings/text%20analysis/themes.pdf.

[16] Thomas J, Harden A. Methods for the thematic synthesis of qualitative research in systematic reviews. BMC Med Res Methodol. 2008;8(1):45. Available from: https://bmcmedresmethodol.biomedcentral.com/track/pdf/10.1186/1471-2288-8-45?site=bmcmedresmethodol.biomedcentral.com. 10.1186/1471-2288-8-45 PMC2478656 18616818

[17] Loke AY, Davies L, Li S. Factors influencing the decision that women make on their mode of delivery: the health belief model. BMC Health Serv Res. 2015;15:274. Available from: https://www.ncbi.nlm.nih.gov/pmc/articles/PMC4506759/pdf/12913_2015_Article_931.pdf. 10.1186/s12913-015-0931-z PMC4506759 26188472

[18] Mselle LT, Moland KM, Mvungi A, Evjen-olsen B, Kohi TW. Why give birth in health facility ? Users ’ and providers ’ accounts of poor quality of birth care in Tanzania. BMC Health Services Research. 2013;13:174. Available from https://bmchealthservres.biomedcentral.com/track/pdf/10.1186/1472-6963-13-174?site=bmchealthservres.biomedcentral.com. 10.1186/1472-6963-13-174 PMC3654954 23663299

[19] Nanjala M, Wamalwa D (2012). Determinants of male partner involvement in promoting deliveries by skilled attendants in Busia. Kenya Glob J Health Sci.

[20] Bedford J, Gandhi M, Admassu M, Girma A (2013). “A normal delivery takes place at home”: a qualitative study of the location of childbirth in rural Ethiopia. Matern Child Health J.

[21] Bohren MA, Hunter EC, Munthe-Kaas HM, Souza J, Vogel JP, Gülmezoglu A. Facilitators and barriers to facility-based delivery in low- and middle-income countries: a qualitative evidence synthesis. Reprod Health. 2014;11(1):71. Available from: https://reproductive-healthjournal.biomedcentral.com/track/pdf/10.1186/1742-4755-11-71?site=reproductive-health-journal.biomedcentral.com. 10.1186/1742-4755-11-71 PMC4247708 25238684

[22] Mrisho M, Schellenberg JA, Mushi AK, Obrist B, Mshinda H, Tanner M (2007). Factors affecting home delivery in rural Tanzania. Trop Med Int Heal.

[23] Lori JR. Cultural Childbirth Practices, beliefs and Traditions in Liberia. The University of Arizona; 2009. 1–90 p.

[24] White D, Dynes M, Rubardt M, Sissoko K, Stephenson R (2013). The influence of intra familial power on maternal health care in Mali: perspectives of women, men and mothers-in-law. Int Perspect Sex Reprod Health.

[25] Mahiti GR, Kiwara AD, Mbekenga CK, Hurtig A-K, Goicolea I. “We have been working overnight without sleeping”: traditional birth attendants’ practices and perceptions of post-partum care services in rural Tanzania. BMC Pregnancy Childbirth. 2015;15(1):8. Available from: https://www.researchgate.net/publication/272076685_We_have_been_working_overnight_without_sleeping_Traditional_birth_attendants'_practices_and_perceptions_of_postpartum_care_services_in_rural_Tanzania. 10.1186/s12884-015-0445-z PMC4324777 25643622

[26] Danforth E, Kruk P, Mbaruku G, Galea S (2009). Household decision – making about delivery in health facilities: evidence from Tanzania. J Health Popul Nutr.

[27] Magoma M, Requejo J, Campbell O, Cousens S, Filippi V. High ANC coverage and low skilled attendance in a rural Tanzanian district: a case for implementing a birth plan intervention. BMC Pregnancy Childbirth. 2010. https://bmcpregnancychildbirth.biomedcentral.com/track/pdf/10.1186/1471-2393-10-13?site=bmcpregnancychildbirth.biomedcentral.com. 10.1186/1471-2393-10-13 PMC2850322 20302625

